# Smart Brain Care – A novel approach to health care of mild cognitive impairment and mild dementia

**DOI:** 10.1093/eurpub/ckac131.030

**Published:** 2022-10-25

**Authors:** A-K Schwientek, L Wolski, L Riedl, T Grimmer

**Affiliations:** Center for Cognitive Disorders, Technical University of Munich, School of Medicine, Munich, Germany

## Abstract

**Background:**

Dementia is a huge burden. Trends for the aging European nations indicate a significant increase of people with dementia. Prevention and early detection are the key elements to reduce risk factors and to treat reversible causes. In addition, identifying people at the beginning of the disease process is crucial when disease-modifying therapy becomes available.

**Methods:**

We aim to develop a complex intervention to improve the identification, diagnosis, and treatment of people with mild cognitive impairment (MCI) and mild dementia syndromes. Our goal is to reduce the time to diagnosis, raising awareness for MCI and increase patient involvement. First, barriers in pathways of care are determined in a systematic review and refined by conducting focus groups and interviews with primary care physicians (PCPs), specialists and experts in the field. A first theory about intervention components and how they interact are developed and will be further tested and adapted through development phase, feasibility and piloting.

**Results:**

The first blueprint of Smart Brain Care addresses three identified key barriers for an improved care of MCI and mild dementia syndromes (communication, skills and awareness) by three interventions (1-3), all linked and accessible through an e-database: An online platform (1) for exchange between PCPs, specialists and experts, (2) a screening tool, diagnosis-algorithm and training for PCPs as well as (3) information materials (e.g., fact sheets, short videos) separately accessible for patients.

**Conclusions:**

There is still low awareness about MCI under health care providers and the general population. A lack of knowledge, cooperation strategies and guidance seem to play an important role for the management of MCI and mild dementia syndromes.

**Key messages:**

• We need to increase focusing on the identification of people with MCI and mild dementia syndromes.

• Call for a structured diagnosis and treatment regimen to support primary care physicians as key players in the management of MCI and mild dementia syndromes.

